# High throughput diagnostics and dynamic risk assessment of SARS-CoV-2 variants of concern

**DOI:** 10.1016/j.ebiom.2021.103540

**Published:** 2021-08-12

**Authors:** Alfredo Maria Gravagnuolo, Layla Faqih, Cara Cronshaw, Jacquelyn Wynn, Paul Klapper, Mark Wigglesworth

**Affiliations:** a Medicines Discovery Catapult, Lighthouse Labs Network, Alderley Park, Mereside, Alderley Edge, Cheshire SK10 4TG, United Kingdom; b Department of Health and Social Care, 39 Victoria Street, London SW1H 0EU, United Kingdom; c The University of Manchester, Oxford Rd, Manchester M13 9PL, United Kingdom; d Discovery Sciences, Biopharmaceuticals R&D, AstraZeneca, Alderley Park, Mereside, Alderley Edge, Cheshire SK10 4TG, United Kingdom

**Keywords:** Real-time tracking, Epidemiological surveillance, Epidemiological investigation, Mass Testing, VOC-202012/01,B.1.1.7

## Abstract

**Background:**

The rise of new SARS-CoV-2 variants worldwide requires global molecular surveillance strategies to support public health control. Early detection and evaluation of their associated risk of spreading within the population are pivotal.

**Methods:**

Between April 2020 and February 2021, the UK Lighthouse Labs Network at Alderley Park tested more than eight million nose and throat swab samples for the presence of SARS-CoV-2, via PCR. The assay targeted three genomic regions of the virus: *N*, Orf1ab and *S*. Whole-genome next-generation sequencing was used to confirm positive PCR results. Positive results were mapped using the postal district origin of samples to allow real-time tracking of the spread of a new variant through the UK.

**Findings:**

In mid-November 2020, the assay identified an increasing number of *S* gene negative, *N* and Orf1ab positive samples. Whole-genome sequencing demonstrated that the loss of *S* gene detection was due to the appearance of a SARS-CoV-2 lineage (B.1.1.7) designated as Variant of concern (VOC) 202012/01. By the beginning of January 2021, the new SARS-CoV-2 VOC comprised 70% of daily positive samples tested at Alderley Park and ∼98% by the end of February 2021.

**Interpretation:**

The timeline view identified the rapid spread of the new SARS-CoV-2 variant across England during the first three weeks of December. Coupling high-throughput diagnostics and molecular surveillance was pivotal to the early detection of the spread of this variant. The availability of real-time tracking of an emerging variant is an important new tool to inform decision-making authorities for risk mitigation. In a respiratory pandemic, a tool for the timely response to the emergence and spread of a novel variant is vital, even more so when a variant is associated with the enhanced transmission, as has occurred with VOC 202012/01.

**Research in context**

**Evidence before this study**

The VOC202012/01 (lineage B.1.1.7) was initially identified in Kent, England in September 2020. Public Health England (PHE, UK GOV) analysed the Kent cluster on the 8th of December and reported it in a technical briefing on the 21st of December 2020. The spread of this new lineage was not noted until mid-November, when an increasing number of *N* and Orf1ab gene-positive samples were noted to have a failure of *S* gene detection by PCR among samples tested at the Alderley Park and Milton Keynes testing facilities of the Lighthouse Labs Network.

The terms “B.1.1.7”, “B1.1.7”, “N501Y”, “501Y Variant 2”, “VUI-202012/01” (variant under investigation), “20I/501Y.V1” (formerly “20B/501Y.V1”), “501Y.V1”, “Kent cluster” were given to the “Kent variant” before it was designated as “VOC 202012/01” (variant of concern) by PHE, and more recently it was denominated Alpha variant or Alpha VOC. Two online news reports mentioning transmission of “N501Y” and “VUI – 202012/01” were published by the COVID-19 Genomics UK Consortium (COG-UK) and PHE on the 14th December 2020, and an announcement by the World Health Organisation (WHO) appeared via a Disease Outbreak News article (21st Dec). On the 18th of December, a preliminary document by COG-UK (available at Virological.org) first explored the novel lineage; analysis of viral loads and the transmission rate of the variant were discussed at the New and Emerging Respiratory Virus Threats Advisory Group (NERVTAG, UK GOV) meeting with PHE (minutes available on the Knowledge Hub). Following the initial technical briefing (21st Dec), PHE deposited a more extensive laboratory report on the medRxiv preprint server (27th Dec).

Our study commenced in mid-November 2020. We submitted the preliminary results to medRxiv on the 7th January 2021, and posted it online on the 15th January 2021.

**Added value of this study**

For the first time in a pandemic, the temporal and geographical spread of a viral variant was observed in real-time, using data from a single large testing facility, coupled with genomic analysis. The mass testing of samples (more than eight million samples tested from April 2020 till February 2021) allowed a clear indication of the very rapid spread of the variant, which was identified as “of concern” in mid-December 2020.

**Implications of all the available evidence**

Large-scale diagnostics can allow tracking of the spread of new virus variants in real-time and guide time efficient decision making.

Alt-text: Unlabelled box

## 1 Introduction

The numerous novel lineages of SARS-CoV-2 detected worldwide have mobilised global molecular surveillance actions [1], [2], [3], [4], [5], [6], [7]. It is known that the mutation rate of SARS-CoV-2 is lower than other RNA viruses such as the influenza virus, probably due to the virus’ internal proofreading mechanism [8,9]. Furthermore, many of the new SARS-CoV-2 variants do not increase the severity of impact; thus, caution should be used before increasing alert levels [5,[10], [11], [12], [13]]. However, accumulation of mutations over time may change virulence, increase the risk of mortality [14], [15], [16], [17], [18], lead to vaccine immune escape and increase transmissibility [19], [20], [21], [22].

Consequently, timely detection and study of recurrence of these mutations and their impact on pandemic countermeasures, such as vaccination, is pivotal [23,24]. Public Health Control measures based on molecular surveillance aims to tackle multiple risks by: sustaining a systematic and robust vaccination campaign [12,[25], [26], [27], [28], [29]]; helping to monitor the level of hospitalization [30,31], and identifying critical strains in hospitals [16,32]; modulating social measures from the basic mandatory face mask to a more drastic lockdown [33].

The detection of a variant alone does not signify whether the variant will assume importance in the population at risk [5,34]. Mass testing can achieve early detection of variant spread (as shown in the present study), and quantification of viral loads in vaccinated and non-vaccinated cohorts [26,35], making possible a comprehensive risk assessment for the population [19], thus, informing decision makers to undertake balanced and controlled measures [36,37].

The Alderley Park (AP) high-throughput diagnostic facility, a part of the Lighthouse Labs (LHLs) Network, was established on the 18th March 2020 by local volunteers from different companies, universities, research institutes and the UK Government, to test for SARS-CoV-2 infection [38,39]. On the 7th April 2020 AP started screening samples of patients and the capacity increased to 80,000 samples per day. The facility uses the ThermoFisher TaqPath™ COVID-19 test for real-time reverse transcription quantitative polymerase chain reaction (RT-qPCR) detection of SARS-CoV-2 and the assay targets three genomic sequences (ORF1ab, *N* and *S*) to provide reliable detection of SARS-CoV-2 [40].

The LHLs facilities receive samples from across the UK, and sampling at any one site of the LHLs Network can enable an estimate of the UK distribution of SARS-CoV-2 infection. During December 2020, we were able to map the temporal and geographical spread of a new variant from its initial region of identification (South-Est England) through to national distribution of infection, in real-time, using semi-automated data analysis of PCR test results.

## 2 Methods

### 2.1 Sample acquisition

A distribution network load, balanced sample distribution to the Alderley Park facility of the LHLs Network, one of the five major UK centres, on a seven-day schedule. Nose and throat swab samples in virus transport medium collected at a variety of test centres and self-collected home samples were delivered from any area of the UK. Sample tracking data was held centrally in a proprietary database (Edge; Department of Health and Social Care, UK Government). The COVID-19 National Testing Programme had turnaround targets for all sample channels, which were either < 24 h or < 48 h. The majority of samples were processed within these targets and these KPI's were tracked and monitored closely.

Samples were shipped to the Labs to optimise turnaround time and LHLs Network utilisation. Whilst Alderley Park generally received a higher proportion of samples from local sites, it cyclically sampled from all around Britain. Mappings were consistent week to week. The most significant changes came from rare diversions and were more common due to the generally lower number of samples at weekends. A trend worth nothing was that labs received fewer satellite samples at weekends, which may account for a change in positivity on Sunday and Monday.

### 2.2 SARS-CoV-2 testing

Samples were extracted using the MagMAX™ Viral/Pathogen Nucleic Acid Isolation Kit (Thermofisher, Warrington, UK) and Kingfisher Flex extraction platform (ThermoFisher). PCR amplification was carried using the TaqPath™ COVID-19 Combo Kit (Thermofisher) with 384 well format on a Quantstudio Flex 7 System (Thermofisher) and analysed by FastFinder® PCR analysis software (UgenTec, Hasselt, Belgium). FastFinder® PCR analysis software (UgenTec, Hasselt, Belgium) used an artificial intelligence algorithm (AI) and semi-automated analysis to assign Cq value to the amplification curves of the assay targets. In concert with human review of results, FastFinder® assigned positive, negative, inconclusive, or invalid test results based on a clinical decision tree, which considered Cq values of the three assay targets, the internal MS2 control and the positive and negative controls on the PCR plate.

### 2.3 Limit of detection

The limit of detection of the assay was less than 500 dcopies* mL^−1^ (*as determined by droplet digital PCR) of virus transport medium. RT-qPCR is considered the gold standard to detect viral genetic material and it is used as a reference method in molecular diagnostics. Concentrations of viral genome in nose and throat swab samples detectable by PCR typically range between single copy and 10^9^ digital copies per mL of virus transport medium. The upper value corresponded to patients with the highest viral loads at the peak of infection. Limit of detections studies, determined the lowest detectable viral copies per mL^−1^ at which 95% of all replicates tested positive (*N* gene 67 dcopies mL^−1^, 2.7 dcopies per reaction, Orf1ab 125 dcopies mL^−1^, 5 dcopies/reaction; *S* gene 250 dcopies mL^−1^, 10 dcopies/reaction). This means that lower concentrations could still be detected, but the % of false negatives increased above 5% when the concentration of viral copies approached values at or below the LoD. Since the LoD was close to the lower boundary of the range of clinical samples only a minor percentage of false negatives were expected. A recent study, considering samples analyzed at the LHLs Network, has estimated the clinical sensitivity at around 95% [41].

### 2.4 Geographic and temporal linkage

Positive samples, tested at Alderley Park LHLs from the 1st to the 21st of December 2020, with *S* gene not detected, were linked to both the subject postal district (Fig. 2 and supporting information video map), and test region (Table 2) by interrogation of Edge data. Data were limited to numbers (counts) of cases from the Alderley Park data set and not normalized by the total number of positive test results.

### 2.5 Molecular analysis

Residual extracted RNA from SARS-CoV-2 positive samples were stored frozen at -20 °C and transferred to the Wellcome Sanger Institute [42], Cambridge, UK for whole-genome sequencing to support the work of the COVID-19 Genomics UK Consortium, www.cogconsortium.uk [43].

### 2.6 Ethics statement

The Head of Approvals Support of the NHS-Health Research Authority, confirmed that as this study was classified as health surveillance and used existing anonymised samples and data, it did not require specific Research Ethics approval.

### 2.7 Statistics

Since the beginning of April 2020 until February 2021, AP-LHLs uploaded more than eight million test results onto the Limfinity® database (Brooks Lifescience, Chelmsford, MA, USA). The database stored a total number of 7,08,195 positive test results uploaded at hourly intervals from August 2020 until February 2021. Sample randomisation was not applicable to this study. For the purpose of this epidemiological investigation, we included positive cases only if at least two out of three assay target curves were within the limit of detection (Table S1). Indeed, failure of single target detection in other types of positive cases could also be caused by the low number of viral copies, not only by genetic mutations. Out of the total positives in the assessed time, 6,60,395 met these requirement criteria, and we analysed this subset as described in Table 1. Raw data for December is available in Table S2, supplementary appendix.

**Table 1:** Statistical analysis of 660395 positive test results between April 2020 and February 2021.

Abbreviation	Description	Algorithms and metrics
*Pos*	Positive test results	The number of daily positive test results with at least two positive targets within the limit of detection, uploaded into Limfinity® database between 00:00 and 23:59 GMT.
*Pos3*	Three detected SARS-CoV-2 targets	Number of *Pos* with three positive targets at any Cq
*ORTF*	ORF1ab target failure	Number of *Pos* with ORF1ab target detection failure.
*NGTF*	*N*-gene target failure	Number of *Pos* with *N*-gene target detection failure.
*SGTF*	*S*-gene target failure	Number of *Pos* with *S-gene* target detection failure.
*DR*	Day rate	$DR_{Pos3}=\;\frac{Pos3}{Pos}\;\%\;or\;DR_{SGTF}=\;\frac{SGTF}{Pos}\;\%$
*RA*	Five-day Rolling average	$RA_{SGTF}=\frac{SGTF\;(sum\;of\;the\;last\;5\;days)}{5}$
*RR*	Five-day Rolling rate Fig. 1	$RR_{SGTF}=\frac{SGTF\;(sum\;of\;the\;last\;5\;days)}{Pos\;(sum\;of\;the\;last\;5\;days)}\%$
*ORI*	Geographical origin in England Table 2	$ORI_{London}=\frac{SGTF\;(sum\;from\;1^{st}-21^{st}\;Dec\;in\;London)}{SGTF\;(sum\;from\;1^{st}-21^{st}\;Dec\;in\;England)}\%$

Raw data and results of the December analysis are available in Table S2, electronic supplementary appendix.

### 2.8 Role of the funders

The Lighthouse Laboratories Network was generated using funding from the Department of Health and Social Care (DHSC), UK-Gov. The funders did not have any role in study design, data collection, data analyzes, interpretation, or writing of report.

## 3 Results

**Fig. 1:**
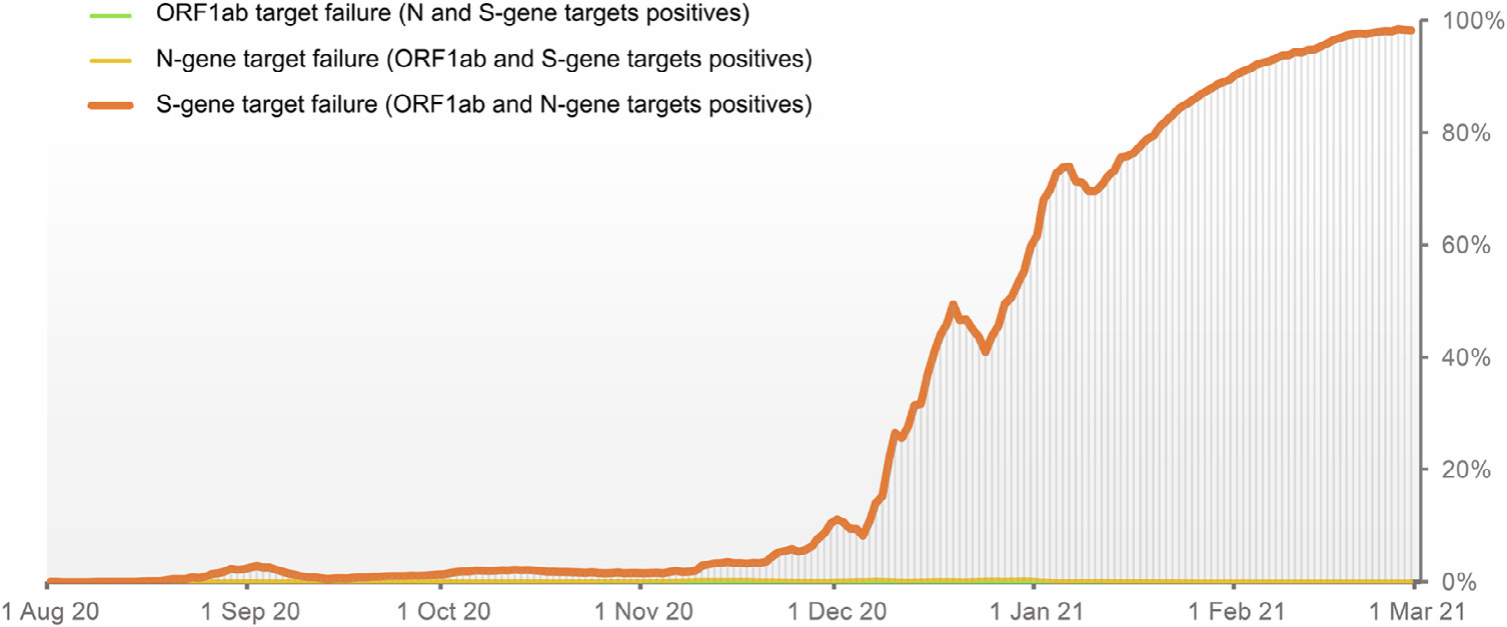
SARS-CoV-2 targets in positive test results. Rise of *S*-gene target detection failure (ORF1ab and *N*-gene positive samples), linked with the SARS-CoV-2 B.1.1.7 (VOC 202012/01). Five-day rolling rate per total number of positives, by date of test results (see *RR*, Table 1). For comparison, failure of ORF1ab and *N*-gene target detection. Samples collected from across England, delivered and tested at Alderley Park, UK. See Table S2 (supplementary appendix) for raw data, *e.g.,* daily number of cases.

**Fig. 2:**
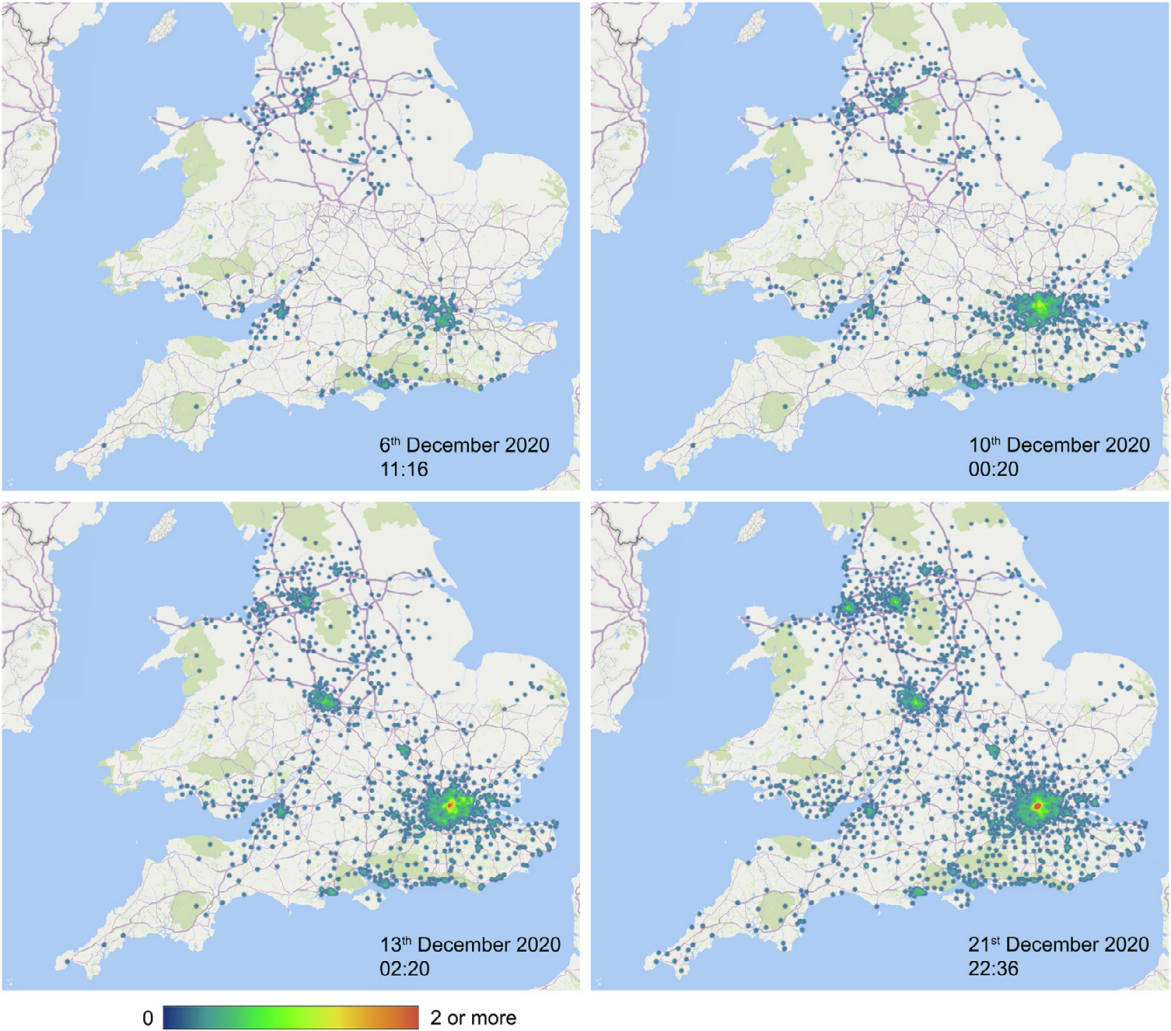
The temporal and geographic spread of VOC 202012/01 in December 2020, seen from the perspective of Alderley Park high-throughput testing facility, Lighthouse Labs Network. The supporting information video map displays detailed spatio-temporal spread. Locations displayed on the Map resulted from the subject postal district and date tested at Alderley Park. Cases displayed on the Map were accumulated over the assessed period in chronological order, generating the heat map. **Heat map**: indicative density of postal districts affected by at least one case of *S*-gene target failure in the areas highlighted by colors (see color bar). Map coverage: 98% of cases listed in Table S2 under column “cases of S-gene target failure” were matched with locations in the assessed time range. Accuracy: 95% of matched locations were displayed with high confidence on the map. The combination of map coverage and accuracy resulted in ∼93% of total cases from Table S2 linked to their locations and, their postal district indicated on the heat map.

At the end of 2020, the proportion of positive specimens tested in England using the ThermoFisher TaqPath™ COVID-19 assay with failure of *S*-gene target detection increased rapidly, rising to more than 70% of positive test results detected within the AP facility by the beginning of January 2021 (Fig. 1, five-day rolling rate). The failure of *S*-gene detection did not significantly alter the clinical validity of the test result, as detection of the alternative genomic targets (ORF1Ab and N) remained robust. However, this non-detection of the *S* gene, allowed surveillance analysis with unprecedented spatio-temporal precision (Fig. 2; and supplementary information video map).

Given that the sample distribution was richer in local (North West England) than distal sites, the spread at high infection rates that we observed at distal sites (*e.g*., London, see Table 2) was slightly underestimated rather than exaggerated. Therefore we believe the conclusions of major conurbations across the UK seeing rapid variant spreading were sound. Those factors, more than a time delay in processing, caused irregularity in the curve in Fig. 1. Considering this bias, the data were consistent with the Office of National Statistics (ONS) dataset [44].

**Table 2:** Geographical origins in England of the cases of *S*-gene target detection failure 1st–21st December 2020.

Region	[%]
London	38.8
South East	30.1
North West	9.9
East of England	7.1
South West	6.7
West Midlands	4.6
East Midlands	2.1
Yorkshire and the Humber	0.7
North East	0.1
Total	100

Samples analyzed at Alderley Park - Lighthouse Labs Network, from 1st–21st December 2020, displayed as fractions of total cases of *S*-gene target failure in the same time range.

In late November 2020, the Sanger Institute confirmed that the increasing number of negative *S*-gene target samples identified by the TaqPath™ COVID-19 test, but positive for the ORF1ab and *N*-gene targets, were due to spike protein mutations characteristic of VOC 202012/01. During December 2020 the number of *S* gene negative samples with ORF1ab and *N*-gene positive increased dramatically and by the end of February 2021 it covered around 98% of the total positive test results (Fig. 1). Specimens were collected from throughout England (Table 2). Linkage of the geographic origin of samples allowed demonstration of the temporal spread of the VOC 202012/01 throughout England and Wales (Fig. 2; and supplementary information video map).

On the 6th of December, cases of *S* gene target detection failure were distributed at low density. By the 10th December, numerous cases were being found throughout the southeast coast of England and in part in the Liverpool and Manchester region. By the 13th December Birmingham, Bristol and London region cases were increasing and by 21st December all major conurbations were affected; this increase was associated with higher levels of patient hospitalization [31].

## 4 Discussion

This report illustrates the spread of a new SARS-CoV-2 variant in December 2020. The combination of high frequency testing and whole-genome sequencing allowed rapid tracking of the SARS-CoV-2 VOC 202012/01.

In mid-November 2020 we noted an increase in the number of SARS-CoV-2 positive samples with failed *S* gene target detection. A percentage of positive samples from the LHLs are referred to the Wellcome Sanger Laboratory Cambridge, UK [42], the main site of the COVID-19 Genomics UK (COG-UK) consortium for whole-genome sequencing [43], currently providing the Global Initiative on Sharing Avian Influenza Data (GISAID) with around 50% of its genomic data [29]. The samples with failure to detect *S* gene amplification were identified to have a deletion of six nucleotides in the *S*-gene (in the PCR probe binding region) forming a new strain of virus (SARS-CoV-2 lineage B.1.1.7.) later designated as VOC 202012/01. The mutations in the *S* gene caused the loss of two amino acids of the Spike protein at positions 69 and 70 (ΔH69/ΔV70) [1].

In late 2020 mutations identified in VOC 202012/01 resulted in the rapid spread of the variant throughout England [1,34,35,[45], [46], [47], [48], [49], [50], [51]]. Key mutations included: N501Y, a key contact residue in the receptor-binding domain, alteration of which is believed to lead to an increase in ACE2 receptor affinity [52]; P681H, one of the four residues that creates a furin cleavage site between the S1/S2 spike protein subunits, promoting the entry of the virus into the respiratory epithelial cells [1].

The net effect of these *S* gene mutations is thought to improve the ability of the virus to attach to the ACE2 cellular receptor, facilitating the infection of epithelial cells leading to the production of virus with greater transmissibility [20,21]. Indeed, the reproduction number of the VOC 202012/01 has been estimated to be 43–90% (95% credible intervals: 38 to 130%) higher than other “non-VOC” pre-existing variants. Further mutations in combination with the existing ones can generate variants with novel properties [50,53].

An early analysis of the risk of mortality carried out by the New and Emerging Respiratory Virus Threats Advisory Group (NERVTAG, UK Government) and independently by several other organisations, inferred a potentially increased disease severity in patients infected with the VOC 202012/01 with the respect to the other circulating variants [14], [15], [16], [17], [18]. A matched cohort study of the University of Exeter on people who tested positive through the LHLs Network, estimated that individuals infected with the VOC were 64% (95% confidence interval, 32 to 104%) more likely to die when compared with equivalent patients infected with circulating non-VOC, in close agreement with data of Public Health England (PHE).

In addition to the aforementioned increased transmissibility and hazard of death, the hazard of hospital admission is critical for the prediction of the burden of the healthcare system following the spread of a new variant. A national study has estimated the risk of hospitalization to be 52% (95% confidence interval, 47 to 57%) higher with the VOC 202012/01 then with the pre-existing variants, within 14 days from the first positive SARS-CoV-2 test [54].

The serendipitous observation of the failure of *S* gene target detection when using the SARS-CoV-2 TaqPath™ COVID-19 Combo multiplex assay allowed real-time tracking of the spread of the variant using PCR. This was a critical new epidemiological tool to support decisions concerning lockdown based on the very rapid spread of the variant from the South-East to major population centres seen between the 6th and 13th December (Fig. 2).

The distribution of VOC 202012/01 cases showed an initially higher burden in London, South East, parts of the North West, South West regions and West Midlands. While the sample collection and distribution network load balancing does not allow the same number of samples to be delivered consistently from each geographic area, the data does provide some indication of the national geographic spread of the variant with time.

As the numbers of collected samples were not equal from each region, a low detection rate cannot be considered conclusive proof of the variant not being present. Likewise, we could not determine if the three temporary drops in the VOC % in December/January (Fig. 1) were caused by variation in geographical sample collection. However, when viewed as a percentage of samples from different regions in the whole period of interest (first three weeks of December), the data showed a clear geographical bias within the overall sample set (Table 2).

Data from epidemiological tracing supported a renewed lockdown before the Christmas period. The proportion of positive specimens tested using the ThermoFisher TaqPath™ COVID-19 assay with failure of *S*-gene target detection (linked to VOC 202012/01) increased to around 98% of positive test results detected within the facility by the end of February 2021, clearly identifying the VOC 202012/01 as the dominant strain in the UK.

The data illustrated the benefit of coupling large scale testing to genomic analysis. While viral variants continue to be identified, a high level of genomic surveillance provides a rapid means of evaluating the significance of an individual variant and early indications concerning viral phenotypes.

The limitation of this method of surveillance is the use of a single RT-qPCR assay to rapidly identify variant spread. Mutations can occur in any part of the viral genome, whereas alteration in the primer/probe binding regions of the RT-qPCR is required for the confirmation of the presence of a variant. Indeed, if the viral gene mutated is in a part not targeted by the RT-qPCR, detection of the variant will be missed [13].

Secondly, more than one viral strain can produce *S* gene detection failure in the TaqPath assay [1,50], thus, detection of *S* gene failure required secondary identification of the actual variant. Rapid testing using single nucleotide polymorphism specific PCR primers [27] can provide faster detection of variants, but are limited to the identification of known variants. Coupling mass testing with genomic surveillance thus remains the only method of identification of as yet ‘unknown’ variants of concern.

Surveillance of the spread of infections has previously relied upon sentinel surveillance sites (with sampling limited to small populations), and surveillance of hospital and general healthcare reports of infection to determine spread within the population [32]. This process is neither comprehensive nor timely [7,55]. Publicly available databases and bioinformatics initiatives [56] (*e.g.* GISAID, PANGO, Pangolin or Nextstrain) [4,[57], [58], [59], [60]], aim to provide real-time surveillance but in reality take between two and six weeks for sequencing and analysis [50]. The advent of high levels of testing coupled with extensive genomic surveillance [6], ushers in a new era of epidemiological surveillance.

## Contributors

AMG is a nanobiotechnology and data scientist; LF is an expert in clinical virology; CC is a Government relationship manager; JW is an operations manager and quality assurance specialist; PK is a professor of clinical virology and clinical adviser to NHS Track and Trace; MW is a director of high throughput screening in a molecular diagnostics setting.

AMG, JW, conceived the idea; MW led the funding acquisition; LF, MW, PK supervised the project; CC, LF, AMG, MW and JW provided the data and resources; AMG, LF, PK, MW performed the formal data analysis; LF, AMG and MW drafted the first manuscript; AMG, PK, MW and LF edited the manuscript; all authors critically reviewed and approved the final manuscript. AMG is the guarantor.

## Declaration of Competing Interest

The authors have nothing to disclose.

## Appendix Supplementary materials

Video S1. Temporal and geographic spread of VOC 202012/01 seen in December 2020: videomap. Image, video 1

Image, application 2
