# Supplementary material for: High throughput diagnostics and dynamic risk assessment of SARS-CoV-2 variants of concern
Source: eBioMedicine. 2021 Aug 12;70:103540. doi: 10.1016/j.ebiom.2021.103540 (PMC8358312; doi:10.1016/j.ebiom.2021.103540)
Supplement: Supplementary file 2 [file mmc2.docx]

Supplementary Appendix

High Throughput Diagnostics and Dynamic Risk Assessment of Variants of Concern

Alfredo Maria Gravagnuolo, PhD^1^ Layla Faqih, PhD^1^ Cara Cronshaw^2^ Jackie Wynn^1^ Paul Klapper, PhD^2,3^ Mark Wigglesworth, PhD^1,4^

^1^ Medicines Discovery Catapult, Lighthouse Labs, Alderley Park, Mereside, Alderley Edge, Cheshire, SK10 4TG, United Kingdom

^2^ Department of Health and Social Care, 39 Victoria Street, London, SW1H 0EU, United Kingdom.

^3^ The University of Manchester, Oxford Rd, Manchester, M13 9PL, United Kingdom

^4^ Discovery Sciences, Biopharmaceuticals R&D, AstraZeneca, Alderley Park, Mereside, Alderley Edge, Cheshire, SK10 4TG, United Kingdom

**Correspondence to:**

Dr Mark Wigglesworth

Director Hit Discovery, Discovery Sciences, Biopharmaceuticals R&D, AstraZeneca, Alderley Park, Mereside, Director Hit Discovery, Alderley Edge, Cheshire, SK10 4TG, United Kingdom Email: [mark.wigglesworth@md.catapult.org.uk](mailto:mark.wigglesworth@md.catapult.org.uk).

**Table S1. Inclusion criteria for positive test results in this study**.

Cut-off values of Cq for positive test results (greater than 500 digital copies mL^-1^ of virus transport medium).

| **At least two targets** | | |
| --- | --- | --- |
| ORF1ab Cq < 31.6 | N gene Cq < 30.1 | S gene Cq < 31.6 |

**Table S2. December raw data of Alderley Park, Lighthouse Labs Network.**

| Date tested at Alderley Park Lighthouse Labs | **TOT numbers of positive Test Results**, (including cases that did not meet criteria of  Table 2). | ***Pos***  **Positive Test Results (TRs)**  (only cases that met criteria of  Table 2) | ***Pos3***  Cases of  **three detected SARS-CoV-2 targets**  in positive TRs | ${DR}_{Pos3}$  ***Day Rate*** *per positive TRs* | ***ORTF***  Cases of  **ORF1ab target**  **failure**  in positive TRs  (N and  S-genes targets  positives) | ***NGTF***  Cases of  **N-gene target**  **failure**  in positive TRs  (ORF1ab and  S-gene targets positives) | ***SGTF***  Cases of  **S-gene target**  **failure**  in positive TRs  (ORF1ab and  N-gene target positives) | ${RA}_{SGTF}$  *Five-day* ***Rolling*** ***Average*** | ${DR}_{SGTF}$  ***Day Rate*** *per positive TRs* | ${RR}_{SGTF}$  *Five-day* ***Rolling Rate*** *per positive TRs* |
| --- | --- | --- | --- | --- | --- | --- | --- | --- | --- | --- |
| 27/11/2020 | 3195 | 2837 | 2571 | 90.6% | 1 | 7 | 258 | by date of test result | 9.1% | by date of test result |
| 28/11/2020 | 2552 | 2258 | 1972 | 87.3% | 0 | 3 | 283 |  | 12.5% |  |
| 29/11/2020 | 1912 | 1732 | 1599 | 92.3% | 0 | 3 | 130 |  | 7.5% |  |
| 30/11/2020 | 3076 | 2801 | 2445 | 87.3% | 0 | 12 | 344 |  | 12.3% |  |
| 01/12/2020 | 2917 | **2662** | 2305 | 86.6% | 0 | 9 | 348 | 273 | 13.1% | 11.1% |
| 02/12/2020 | 2475 | **2280** | 2144 | 94.0% | 0 | 3 | 133 | 248 | 5.8% | 10.6% |
| 03/12/2020 | 2278 | **2102** | 1959 | 93.2% | 0 | 9 | 134 | 218 | 6.4% | 9.4% |
| 04/12/2020 | 1814 | **1664** | 1540 | 92.5% | 0 | 7 | 117 | 215 | 7.0% | 9.3% |
| 05/12/2020 | 2928 | **2697** | 2481 | 92.0% | 0 | 12 | 204 | 187 | 7.6% | 8.2% |
| 06/12/2020 | 2350 | **2147** | 1562 | 72.8% | 0 | 9 | 576 | 233 | 26.8% | 10.7% |
| 07/12/2020 | 1832 | **1706** | 1279 | 75.0% | 1 | 9 | 417 | 290 | 24.4% | 14.0% |
| 08/12/2020 | 2174 | **2012** | 1763 | 87.6% | 0 | 6 | 243 | 311 | 12.1% | 15.2% |
| 09/12/2020 | 3968 | **3669** | 2464 | 67.2% | 0 | 10 | 1195 | 527 | 32.6% | 21.5% |
| 10/12/2020 | 4537 | **4222** | 3002 | 71.1% | 0 | 11 | 1209 | 728 | 28.6% | 26.5% |
| 11/12/2020 | 4205 | **3954** | 3027 | 76.6% | 1 | 9 | 917 | 796 | 23.2% | 25.6% |
| 12/12/2020 | 4538 | **4281** | 2840 | 66.3% | 1 | 9 | 1431 | 999 | 33.4% | 27.5% |
| 13/12/2020 | 4725 | **4416** | 2689 | 60.9% | 0 | 16 | 1711 | 1293 | 38.7% | 31.5% |
| 14/12/2020 | 5383 | **5073** | 3367 | 66.4% | 0 | 21 | 1685 | 1391 | 33.2% | 31.7% |
| 15/12/2020 | 5867 | **5522** | 2694 | 48.8% | 0 | 23 | 2805 | 1710 | 50.8% | 36.8% |
| 16/12/2020 | 5230 | **4912** | 2706 | 55.1% | 0 | 15 | 2191 | 1965 | 44.6% | 40.6% |
| 17/12/2020 | 5891 | **5614** | 2765 | 49.3% | 0 | 20 | 2829 | 2244 | 50.4% | 43.9% |
| 18/12/2020 | 4768 | **4523** | 2260 | 50.0% | 0 | 17 | 2246 | 2351 | 49.7% | 45.8% |
| 19/12/2020 | 5289 | **4984** | 2420 | 48.6% | 1 | 16 | 2547 | 2524 | 51.1% | 49.4% |
| 20/12/2020 | 3084 | **2915** | 2024 | 69.4% | 0 | 7 | 884 | 2139 | 30.3% | 46.6% |
| 21/12/2020 | 4225 | **4002** | 2210 | 55.2% | 0 | 12 | 1780 | 2057 | 44.5% | 46.7% |
| 22/12/2020 | 3638 | **3439** | 1942 | 56.5% | 0 | 20 | 1477 | 1787 | 42.9% | 45.0% |
| 23/12/2020 | 3694 | **3452** | 1934 | 56.0% | 0 | 24 | 1494 | 1636 | 43.3% | 43.5% |
| 24/12/2020 | 4098 | **3781** | 2202 | 58.2% | 0 | 14 | 1565 | 1440 | 41.4% | 40.9% |
| 25/12/2020 | 4276 | **3992** | 2130 | 53.4% | 0 | 9 | 1853 | 1634 | 46.4% | 43.8% |
| 26/12/2020 | 4808 | **4504** | 2152 | 47.8% | 0 | 16 | 2336 | 1745 | 51.9% | 45.5% |
| 27/12/2020 | 4898 | **4569** | 1743 | 38.1% | 0 | 21 | 2805 | 2011 | 61.4% | 49.5% |
| 28/12/2020 | 3778 | **3500** | 1748 | 49.9% | 0 | 27 | 1725 | 2057 | 49.3% | 50.5% |
| 29/12/2020 | 3407 | **3169** | 1403 | 44.3% | 0 | 13 | 1753 | 2094 | 55.3% | 53.1% |
| 30/12/2020 | 5667 | **5293** | 2251 | 42.5% | 0 | 22 | 3020 | 2328 | 57.1% | 55.3% |
| 31/12/2020 | 6842 | **6307** | 1966 | 31.2% | 0 | 17 | 4324 | 2725 | 68.6% | 59.7% |
| **Grand total (December)** | **125584** | **117363** | **68972** |  | **4** | **433** | **47954** |  |  |  |
| **Rate**  **(December)** |  | **100%** | **58.77%** |  | **0.0%** | **0.37%** | **40.86%** |  |  |  |
| **Chart** |  |  |  |  |  |  | Figure 2 |  |  | Figure 1 |
